# The Influence of the Annular Nozzle’s Structural Parameters on Powder Stream Convergence for Laser-Directed Energy Deposition

**DOI:** 10.3390/ma18092055

**Published:** 2025-04-30

**Authors:** Bobo Li, Weiyi Wang, Donglai Li, Zong Liu, Yuhang Ren, Yushi Wang, Guang Yang

**Affiliations:** 1 College of Mechatronics Engineer, Shenyang Aerospace University, Shenbei District Daoyi South Street, Shenyang 110136, China; l_ibobo@163.com (B.L.); 13898778805@163.com (W.W.); 15065355661@163.com (Z.L.); yuhang@sau.edu.cn (Y.R.); wysneu@163.com (Y.W.); 2 Key Laboratory of Rapid Development & Manufacturing Technology for Aircraft, Ministry of Education, Shenyang Aerospace University, Shenyang 110136, China; 15630607106@163.com

**Keywords:** laser-directed energy deposition, annular nozzle, waist diameter, powder convergence, structural optimization, resource efficiency

## Abstract

Laser-directed energy deposition (L-DED) technology is increasingly used in the manufacturing industry, in which the powder convergence of the feeding nozzle affects the accuracy and quality of additive manufacturing parts. However, there is nothing in the literature that gives a comprehensive optimization scheme for the powder feeding structure of the ring nozzle. In order to investigate the effect of the internal structure for the annular nozzle on powder convergence, in this paper, finite element analysis models for the annular nozzle are established, and the Lagrangian–Eulerian method is used to analyze the influence of different outlet shapes, powder feeding inclination angles, outlet gaps, and inlet shapes of annular nozzles on the powder convergence. The results indicate that the parallel outlet shape is more suitable for the annular nozzle, and the waist diameter of the powder stream decreases gradually with the decrease in the power feeding inclination angle and the outlet gap. The inlet shape of the powder storage chamber plays a guiding role in the moving direction of the powder particles; when the powder feeding inclination angle is 17° and the outlet gap is 0.5 mm, the waist diameter of the powder stream is reduced by 49%. In addition, the effects of particle size, carrier gas rate, and laser shielding gas on the powder convergence are also studied, and the results indicate that, with the reduction of particle size and carrier gas rate, the powder convergence can be effectively improved, and the waist diameter of powder stream becomes 1.42 mm. With the increase in the laser shielding gas rate, the powder convergence position moves down. The research results provide a basis for the structural optimization to design a high-convergence annular nozzle for the laser additive manufacturing process.

## 1. Introduction

**Figure 1:**
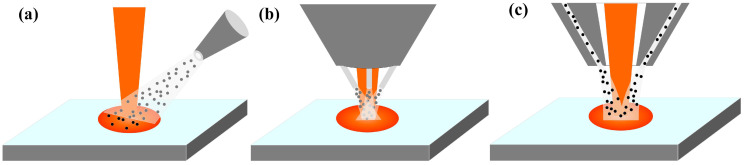
Different types of powder feeding nozzle: (**a**) Off-axial nozzle. (**b**) Discrete coaxial nozzle. (**c**) Annular coaxial nozzle.

Laser-directed energy deposition (L-DED) technology has been gradually becoming mature in recent years, particularly when compared to the traditional processing of titanium alloy and the difficult processing of other metal materials, demonstrating significant processing advantages [1,2,3,4]. This technology improves the limitations of traditional part design [5] and can also be used to repair some defects of parts and extend the service life of parts [6,7,8]. In L-DED forming, the spatial distribution of powder flow concentration directly determines the deposition efficiency, dimensional accuracy, and forming quality [9,10,11]. The waist diameter of the powder flow directly reflects the convergence property of the powder. Having good powder convergence ability can ensure that more powder enters the molten pool within the same period of time, improving the forming efficiency. Meanwhile, it helps to reduce the possible defects inside the workpiece and the surface roughness, thus enhancing the forming quality. One of the important ways of improving the convergence of powder is the optimization of the structure of the powder feeding nozzle. At present, there are three types of powder feeding nozzles widely used, which are off-axis nozzles, discrete coaxial nozzles, and annular coaxial nozzles [12], as shown in Figure 1.

Off-axis nozzles are difficult to manufacture by multidirectional deposition due to their limited structure, so discrete coaxial nozzles and annular coaxial nozzles are more widely used [13]. By means of experimental comparison, Zhong et al. [14] analyzed the powder flow morphology and the corresponding sediment morphology of the discrete coaxial nozzle and the annular coaxial nozzle; they showed that the higher speed of powder particles emitted from the annular nozzle makes more powder have enough kinetic energy to pass through the surface tension of a molten pool, the height of the track printed by the annular nozzle is higher, and the track has a larger cross-sectional area. Therefore, annular nozzles have higher powder utilization. Gao et al. [15] used finite element analysis to compare the powder flow characteristics of two coaxial nozzles: under the same conditions, the powder flow divergence angle of the annular nozzle is smaller, and the powder mass concentration in the convergence plane is higher, which indicates that the powder convergence of the annular nozzle is better. However, the internal structure of annular nozzles is more complex, and the influence of internal structural characteristics on powder flow is one of the important steps in designing annular nozzles.

In order to improve the ability of annular nozzles to converge powder, many scholars have conducted a lot of research on the influence of the internal structure of annular nozzles on powder convergence. By combining simulations and experiments, Pan et al. [16] found that controlling the powder delivery channel of an annular nozzle could effectively change the convergence effect of the powder flow and that a suitable nozzle angle is conducive to the powder convergence and improves the mass concentration of the powder flow center. And the external protective gas output structure can be added to the outside of the bottom end of the nozzle, which helps to produce an ideal powder flow shape and improve powder utilization. However, more structural parameters were not considered to optimize the powder feeding structure and improve the powder convergence. In addition, some scholars proposed to design a variety of groove patterns on the outer cone face of the part and, through a simulation experiment, it is found that the groove structure with appropriate structural dimensions can reduce the tangential velocity component of powder particles to a certain extent, restrict the movement direction of powder particles, and improve the convergence ability of powder particles [17,18]. Some scholars added the rectifier tube structure to the inner upper end of the annular nozzle, and the powder particles entering the nozzle will collide several times when passing through the structure, thus weakening the tangential velocity component of the powder particles, making the direction of the powder movement parallel to the wall, so as to improve the convergence effect of the powder [18,19,20,21,22]. Nogdhe et al. [23] designed the outer shielding gas structure on the outside of the nozzle to help achieve higher powder utilization under the appropriate external shielding gas flow. However, there are few articles that give a complete description of the design and optimization process of annular nozzles.

In addition to the structural influence of the nozzle, the impact of other powder feeding factors on the convergence of powder in the annular nozzle cannot be overlooked. Some scholars [24,25] conducted simulation analysis and discovered that the collision behavior of powder particles inside the nozzle and the spatial distribution of powder flow are influenced by the applied powder particle diameter and elastic recovery coefficient. During the L-DED deposition process, the powder particles rebound when hitting the surface of the substrate, and some of the powder particles absorb the laser heat and adhere to the lower end of the nozzle. Zekovic et al. [26] demonstrated through simulation analysis and experimentation that a shorter working distance of the nozzle, particularly with laser power exceeding 400 W, increases the likelihood of powder particles adhering to the lower section of the nozzle. Laser shielding gas is used to block some of the powder particles from bouncing back into the laser channel and contaminating the optics lens. In order to analyze the influence of laser shielding gas on powder convergence, Jardon et al. [27] used high-speed cameras to photograph the morphology of powder flow under different laser shielding gas speeds and analyzed different optical images, finding that the powder convergence decreased with the increase in laser shielding gas. However, there is little literature discussing the influence of particle size and carrier gas velocity on powder flow in annular nozzles.

Due to the complex powder feeding structure of the annular nozzle, few scholars have been able to study and analyze completely the influence of the characteristic structure parameters of the annular nozzle on the powder convergence, and there is still a gap in the design and optimization of the powder feeding structure of the annular nozzle. Therefore, in order to further improve the convergence effect of laser-directed energy deposition powder particles after ejection from the nozzle and improve the forming efficiency and forming quality of additive manufacturing parts, based on fluid dynamics and finite element analysis, the simulation model of a coaxial annular powder feeding nozzle is established, and the accuracy and reliability of the model and method are verified by verification experiments. In addition, based on the established model, this paper uses numerical simulation to deeply analyze the effects of parameters such as the characteristic structure of the annular powder feeding nozzle (outlet shape, powder feeding inclination angle, outlet gap, and inlet shape), powder particle size distribution range, laser shielding gas, and carrier gas rate on the formed powder stream distribution morphology, the diameter of the powder waist, and the powder mass concentration. The influence law of powder convergence is obtained, which provides a theoretical basis for the structure optimization of powder feeding nozzles and the selection of powder feeding parameters.

## 2. Finite Element Model of the Structure of the L-DED Powder Feeding Nozzle

### 2.1. Annular Nozzle Geometry

**Figure 2:**
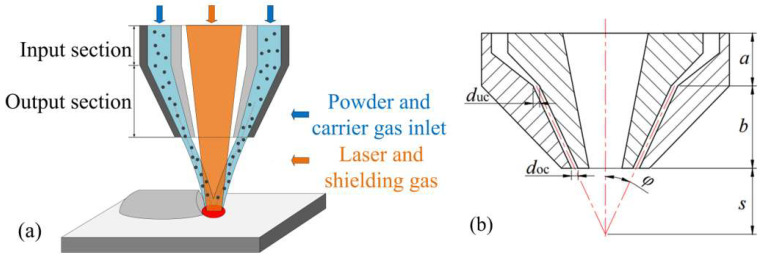
Annular nozzle powder feeding principle and structure diagram: (**a**) Schematic diagram of the division of the powder feeding structure, (**b**) Powder feeding characteristic structure parameters.

In order to further analyze the influence of the powder feeding structure inside the annular nozzle on the powder convergence, the overall structure of the annular nozzle is divided into two parts and discussed successively, as shown in Figure 2.

The internal powder feeding structure of the annular nozzle is divided into the input section (IS) and output section (OS) in the vertical direction, as shown in Figure 2a. Based on this, the characteristic structure of the OS analyzed in this paper includes the size of powder feeding inclination *φ* and gaps *d*_uc_ and *d*_oc_, shown in Figure 2b, and *s* is the nozzle working distance. With the different sizes of gaps duc and doc, the angle between the inner and outer cones of the OS part also changes, and the outlet shape can be divided into three types, which are contraction type (*d*_uc_ > *d*_oc_), parallel type (*d*_uc_ = *d*_oc_), and divergent type (*d*_uc_ < *d*_oc_).

**Figure 3:**
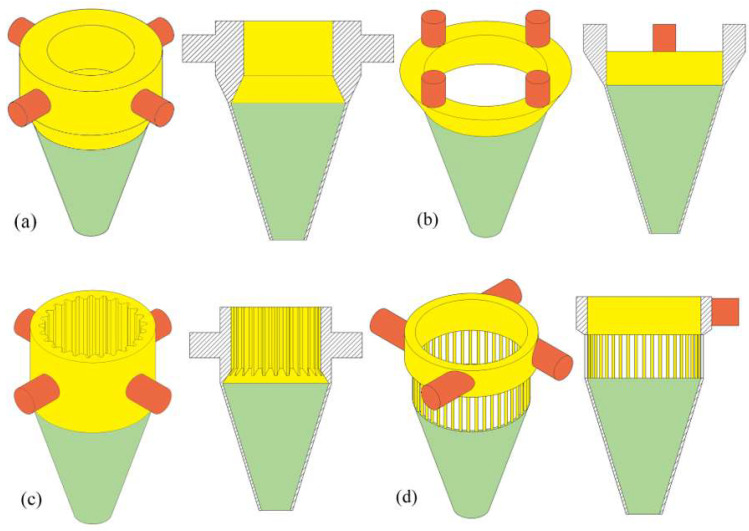
Geometric model of fluid domain with different inlet shapes: (**a**) Powder storage chamber structure, (**b**) The same as the outlet shape, (**c**) Nozzle inner wall with correction straight grooves, (**d**) rectifier tube structure.

The IS of the annular nozzle mainly explores the influence of different inlet shape structures on powder aggregation. This paper establishes an analysis model based on four common inlet shapes, as shown in Figure 3, which are the fluid domain geometric models of the internal powder delivery channels of the four annular nozzles, namely (a) with a powder storage chamber structure [28], (b) with the same outlet shape [29], (c) with a corrected direct-current groove on the inner conical wall of the nozzle [30], and (d) with a rectifier tube structure [19,20].

### 2.2. Simulation Model and Theory

#### 2.2.1. Simulation Model

**Figure 4:**
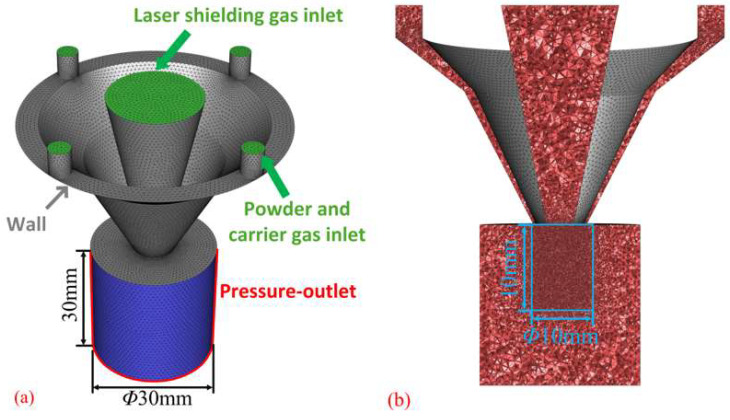
Schematic diagram of boundary conditions of simulation model: (**a**) Boundary condition division, (**b**) Region of encrypted mesh.

During the laser deposition manufacturing process, the nozzle powder delivery channel’s structure, the characteristics of the powder particles, and the gas rate jointly determine the motion state of the powder particles after ejection from the bottom of the nozzle and then affect the powder convergence effect. In order to analyze the influence of the above factors on the powder convergence effect, this paper simulates the powder convergence of the annular nozzle based on the commercial fluid dynamics analysis software Fluent 2020 R2, and Fluent meshing was used to mesh the model of the annular nozzle. The minimum grid size of the whole model is set to 0.2 mm, and the maximum grid size is set to 1 mm. The part shown in Figure 4 is partially encrypted with the grid, and the grid size is unified to 0.2 mm. The overall number of grids is about 2.28 million. In order to simulate the natural environment and characterize the dynamic characteristics of the powder particles after they are ejected from the nozzle, a blue cylindrical area with a diameter of 30 mm was set at the bottom of the powder feeding nozzle. The surrounding boundary is set as the pressure outlet (blue area in Figure 4a), and the inlet plane of the powder feeding structure is set as the velocity inlet boundary condition (green area in Figure 4b). Other dimensional parameters of each part are shown in Table 1.

In the process of simulation calculations to simulate the flow of gas–powder, the following assumptions are made:

1. In the calculation of the simulated powder feeding process, the influence of heat transfer between other heat sources such as laser radiation and the powder particles is not considered, so only the drag force, inertial force, and gravity of the discrete phase are considered, and other forces are ignored.
2. The simulation calculation adopts transient flow analysis, and the time step size is 0.001 s, the number of time steps is 1000, and the convergence accuracy is 10^−3^.
3. Ignoring the bouncing of particles on the substrate, the particle flow is assumed to be a free jet.
4. The gas used is argon. Table 2 shows the specific parameters.
5. The powder particles are spherical, and their particle size distribution adopts the Rosin–Rammler particle size distribution method, and the specific parameters are shown in Table 3.

#### 2.2.2. Continuous Phase Governing Equation

In the process of laser deposition manufacturing powder feeding, when the overall integral number of carrier gas powder particles is less than 10%, the carrier gas is considered as the continuous phase and the powder particles as the discrete phase. The Lagrangian–Eulerian model is employed to solve this model [8,30,31,32,33].

When the powder carrier gas is treated as the continuous phase, the mass conservation equation is:

(1) $$\frac{𝜕\rho }{𝜕t}+\nabla \cdot \left(\rho \vec{v}\right)=0$$

The above Equation (1) is the general form of the mass conservation equation, where *ρ* is the density of the gas; $\vec{v}$ is the velocity of the gas.

The momentum conservation equation of the continuous phase is:

(2) $$\frac{𝜕}{𝜕t}\left(\rho \vec{v}\right)+\nabla \cdot \left(\rho \vec{v}\vec{v}\right)=-\nabla p+\nabla \cdot \left(\overset{̿}{\tau }\right)+\rho \vec{g}+\vec{F}$$

where *p* is static pressure; $\overset{̿}{\tau }$ is the stress tensor; $\rho \vec{g}\;$ and $\vec{F}$ are gravity and external forces, respectively.

The stress tensor can be obtained from Equation (3):

(3) $$\overset{̿}{\tau }=\mu \left[\left(\nabla \vec{v}+\nabla {\vec{v}}^{T}\right)-\frac{2}{3}\nabla \cdot \vec{v}I\right]$$

where *μ* is the molecular viscosity; *I* is the unit tensor; the second item on the right is the effect of volume expansion.

The *k*-*ε* model, which is widely used in the dynamic analysis of L-DED powder particles, was used for numerical simulation. The standard *k*-*ε* model, as the most used model for turbulent flow calculation, was proposed by Launder and Spalding. The standard *k*-*ε* model is an equation based on turbulent kinetic energy (*k*) and dissipation rate (*ε*). The turbulent kinetic energy and dissipation rate can be obtained from the following equation:

(4) $$\frac{𝜕}{𝜕t}\left(\rho k\right)+\frac{𝜕}{𝜕x_{i}}\left(\rho ku_{i}\right)=\frac{𝜕}{𝜕x_{j}}\left[\left(\mu +\frac{\mu_{t}}{\sigma_{k}}\right)\frac{𝜕k}{𝜕x_{j}}\right]+G_{k}+G_{b}-\rho \varepsilon -Y_{M}$$

(5) $$\frac{𝜕}{𝜕t}\left(\rho \varepsilon \right)+\frac{𝜕}{𝜕x_{i}}\left(\rho \varepsilon u_{i}\right)=\frac{𝜕}{𝜕x_{j}}\left[\left(\mu +\frac{\mu_{t}}{\sigma_{\varepsilon }}\right)\frac{𝜕\varepsilon }{𝜕x_{j}}\right]+C_{1\varepsilon }\frac{\varepsilon }{k}\left(G_{K}+G_{3\varepsilon }G_{b}\right)-C_{2\varepsilon }\rho \frac{\varepsilon^{2}}{k}$$

where *G_k_* represents the turbulent kinetic energy generated due to the average velocity gradient; *G_b_* is turbulent kinetic energy generated by buoyancy. *Y_M_* represents the effect of fluctuating expansion on the total dissipation rate in compressible turbulence. *C*_1ε_ = 1.44; *C*_2ε_ = 1.92; *C*_3ε_ = 0.09; *σ*_k_ and *σ*_ε_ are respectively the turbulent Prandtl numbers of the turbulent kinetic energy (*k*) and the dissipation rate (*ε*). *G_k_*, *G_b_*, and *Y_M_* can be determined by the following formula:

(6) $$G_{k}=-\rho u_{i}^{\prime }u_{j}^{\prime }\frac{𝜕u_{j}}{𝜕x_{i}}$$

(7) $$G_{b}=-g_{i}\frac{\mu_{t}}{\rho Pr_{t}}\frac{𝜕\rho }{𝜕x_{i}}$$

(8) $$Y_{M}=2\rho \varepsilon M_{t}^{2}$$

(9) $$M_{t}=\sqrt{\frac{k}{a^{2}}}$$

where *g*_i_ is the component of the gravity vector in the ith direction; *Pr*_t_ is turbulent Prandtl number, the default value of *Pr*_t_ in the standard *k*-*ε* model is 0.85; *M*_t_ is turbulent Mach number; a is the value of sound velocity.

#### 2.2.3. Discrete Phase Governing Equation

The powder particles are regarded as discrete phase. The Lagrange method is used to calculate the force balance on the powder particles and analyze the motion trajectory of the discrete phase particles.

The force balance on the powder particle is equal to the inertia of the particle and the force acting on the particle, which can be determined by the following formula:

(10) $$m_{p}\frac{d\vec{u}}{dt}=m_{p}\frac{\vec{u}-\vec{u_{p}}}{\tau_{r}}+m_{p}\frac{\vec{g}\left(\rho_{p}-\rho \right)}{\rho_{p}}+\vec{F}$$

where *m*_p_ is the particle mass; $\vec{u}$ is the fluid phase velocity; $\vec{u_{p}}$ is particle velocity; *ρ* is the fluid density; *ρ*_p_ is the particle density; $\vec{F}$ is an additional force; $m_{p}\frac{\vec{u}-\vec{u_{p}}}{\tau_{r}}$ is resistance; *τ*_r_ is the relaxation time of the particle, and its formula is as follows:

(11) $$\tau_{r}=\frac{\rho_{p}d_{p}^{2}}{18\mu }\frac{24}{C_{D}Re}$$

where *μ* is fluid viscosity; *d*_p_ is particle diameter; *Re* is relative Reynolds number; *Re* is defined as:

(12) $$Re\equiv \frac{\rho d_{p}\left|{\vec{u}}_{p}-\vec{u}\right|}{\mu }$$

As the interaction between the powder particles and the carrier gas cannot be ignored, momentum transfer between the continuous and discrete phases is required, which can be calculated by the following formula:

(13) $$F=\sum \left(\frac{18\mu C_{D}Re}{\rho_{p}d_{p}^{2}24}\left(u_{p}-u\right)+F_{other}\right){\dot{m}}_{p}∆t$$

where ${\dot{m}}_{p}$ is the mass flow rate of particles; ∆*t* is the time step; *F*_other_ is other interaction force; *C*_D_ is the drag coefficient.

## 3. Experimental Verification

**Figure 5:**
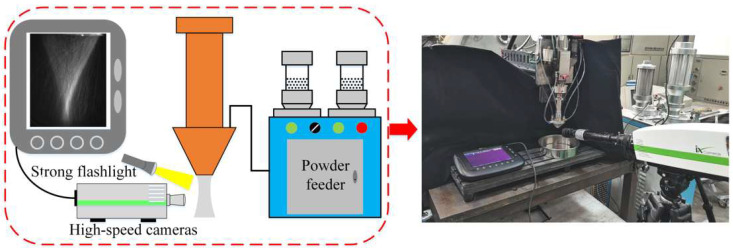
Diagram of experimental equipment.

In order to verify the reliability and accuracy of the results of the numerical model, as well as to provide a favorable basis for the analysis of the influence factors on powder aggregation below, in this paper, an experiment is designed to observe the morphology of powder flow convergence and verify the simulation results, as shown in Figure 5.

In this experiment, coaxial powder feeding additive manufacturing equipment was used. A strong flashlight is used to illuminate the powder flow after ejection from the annular nozzle, and a high-speed camera (Model i-SPEED 3, Dalian Yuanlong Testing Instrument Co., Ltd., Dalian, China) is used to photograph the morphology of the powder flow. During the process of the experiment, the powder particle size range used is 15–53 μm, the material is TC4, the density is 4.5 g/cm^3^, laser shielding gas is set to 1 L/min, and carrier gas flow on the powder feeder is set to 3 L/min. The internal structure parameters of the annular nozzle in the figure are shown in Table 4.

**Figure 6:**
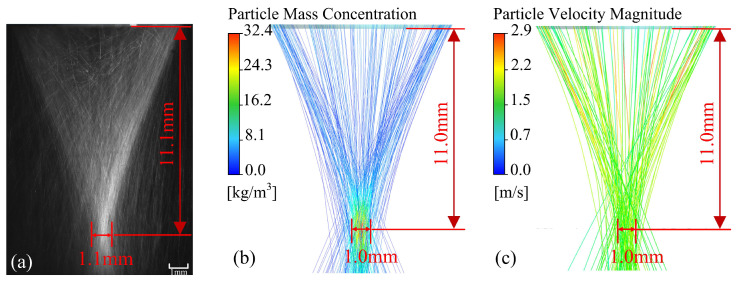
Comparison of experimental and simulation results: (**a**) Experimental results, (**b**) Simulation results colored by concentration, (**c**) Simulation results colored by the magnitude of velocity.

In order to validate the simulation model, the simulation results are compared with the experimental shooting results, as shown in Figure 6. Figure 6a shows the powder flow morphology captured by a high-speed camera, Figure 6b,c depict the trajectories of powder particles colored by concentration and the magnitude of velocity, respectively. The actual location of the powder convergence and the size of the powder waist diameter measured in the experiment are compared with the simulation results; however, since the specific location where the powder concentration reaches 86.5% could not be determined, the powder waist diameter measured in the experiment is inconsistent with the powder waist diameter defined in the subsequent simulation analysis. Good consistency is obtained through quantitative verification and comparison. Indicating that the simulation model can well describe the dynamic behavior of powder particles inside the nozzle and during the convergence process.

## 4. Results and Discussion

### 4.1. Effect of Nozzle Structure on Powder Convergence

The geometric characteristics of the laser-directed energy deposition annular powder feeding nozzle are detailed in Section 2, with the powder feeding structure being divided into the input section (IS) and the output section (OS). The geometric structure features of the output section are reflected in different outlet shapes, powder feeding inclination angle, and outlet gap, while the input section is reflected in the inlet shape. The above factors will affect the convergence effect of powder particles after ejection from the nozzle. In this paper, numerical simulation technology is used to study the influence law of various influencing factors on the powder convergence by the control variable method, and the optimum matching relation of the annular powder feeding nozzle is determined to improve the powder utilization rate.

#### 4.1.1. Outlet Shape

In order to investigate the influence of the outlet shape of the annular powder feeding nozzle on the powder convergence, the geometric parameters of *d*_uc_ and *d*_oc_ are set in this paper as shown in Table 5. In the table, the outlet shapes of Cases 1–4 are contraction type, parallel type, and divergent type, respectively. During the process of numerical simulation, the influence of outlet shape on powder convergence is obtained by changing the size of *d*_uc_ and *d*_oc_, keep the powder feeding inclination angle of 25° and other powder feeding parameters unchanged.

**Figure 7:**
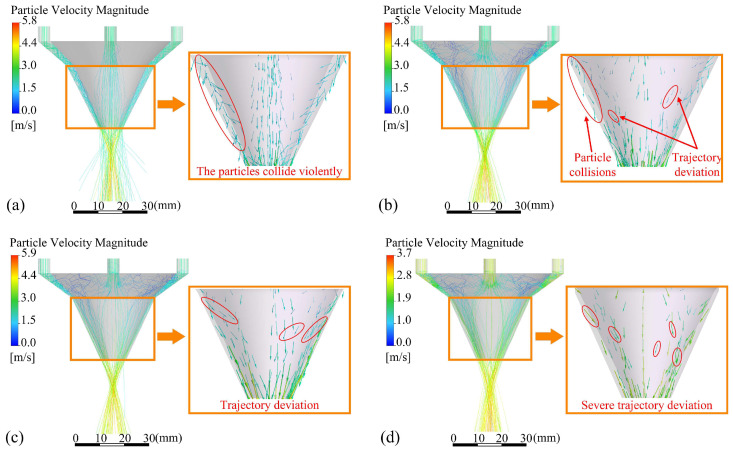
Particle trajectories and velocity vectors of powder in different cases(the vectors of powder particles with collision and obvious tangential velocities are circled in red in the figure): (**a**) Case 1, (**b**) Case 2, (**c**) Case 3, and (**d**) Case 4.

Under the same conditions, powder particles passing through different outlet shapes result in different powder stream morphologies. Figure 7 illustrates the trajectory and velocity vector of the powder particles. The powder particles have multiple collisions with the inner and outer wall surfaces in the contraction type outlet structure. With the increase in *d*_uc_ size, the collision of powder particles is intensified, the convergence of powder becomes worse, and the distribution of powder inside the nozzle is uneven. The divergent type of outlet structure improves the uneven distribution of powder, but the constraining effect of the inner and outer walls of the nozzle on powder particles is reduced due to the increase in *d*_oc_, resulting in the lack of a subsequent movement direction constraint after the powder particles enter the OS part of the nozzle, and the convergence of the powder is not as good as that of the parallel outlet structure.

**Figure 8:**
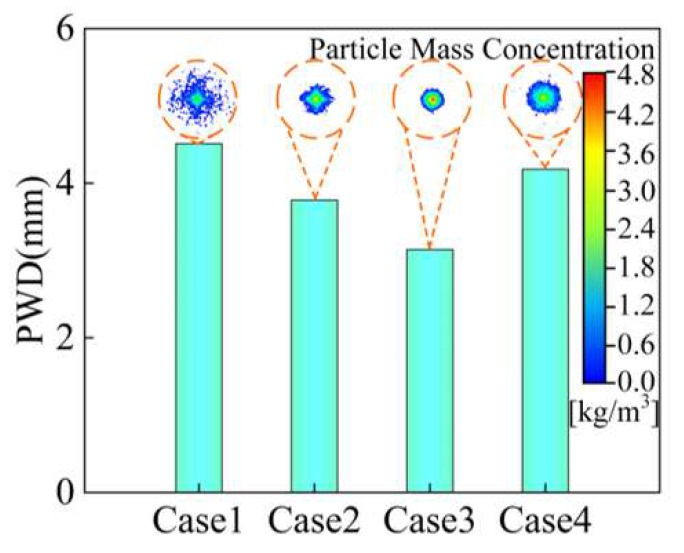
The powder waist diameter and concentration distribution in different cases.

In this paper, the middle point of the convergence plane is the center of the circle and, when the circle completely covers 86.5% of the maximum concentration of powder mass, the diameter of the circle is the powder waist diameter (PWD). Figure 8 shows the waist diameter of the powder stream with different outlet shapes and the powder concentration distribution of the corresponding plane. On the convergence plane, the convergence powder concentration distribution morphology of the contraction type outlet structure presents a “cross shape”, which corresponds to the powder particle movement trajectory in Figure 7, and the powder convergence becomes worse with the increase in *d*_uc_. Among the different outlet structures, the parallel type of outlet structure obtained the minimum waist diameter of the powder stream, which is 3.14 mm, and compared with Case 1, the powder waist diameter is reduced by 30%. Therefore, the parallel type of outlet is the most favorable geometry for powder convergence among the three kinds of outlet structures.

#### 4.1.2. Feeding Inclination Angle

To investigate the influence of nozzle feeding inclination angle *φ* on the powder convergence, the working distance of nozzle 10 mm is taken as the fixed parameter in this paper. A powder feed inclination angle *φ* ranging from 17° to 25° is set as the analysis object to avoid interference with the laser channel structure inside the nozzle if the powder feed inclination angle is too small. The parallel outlet structure is kept unchanged, and the influence of powder feeding inclination angle *φ* on the powder convergence is obtained by changing the size of powder feeding inclination angle *φ.*

**Figure 9:**
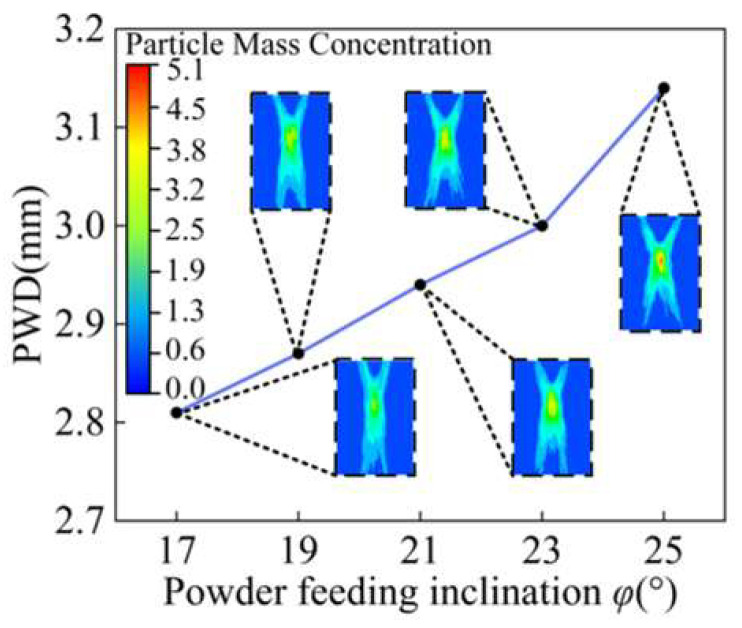
The waist diameter of powder stream with different inclinations *φ*.

The powder waist diameter formed by different powder feeding inclination angles *φ* is shown in Figure 9, and the powder stream cross-section concentration distributions of different angles between 17° and 25° are given respectively. With the decrease in the powder feeding inclination angle *φ*, the distribution of powder particles in the feeding process is more uniform, and the waist diameter of the converging powder stream is smaller. When the feeding inclination angle *φ* is 17°, the minimum waist diameter of the powder stream is 2.81 mm, which is 10.5% lower than that when the feeding inclination angle *φ* is 25°.

**Figure 10:**
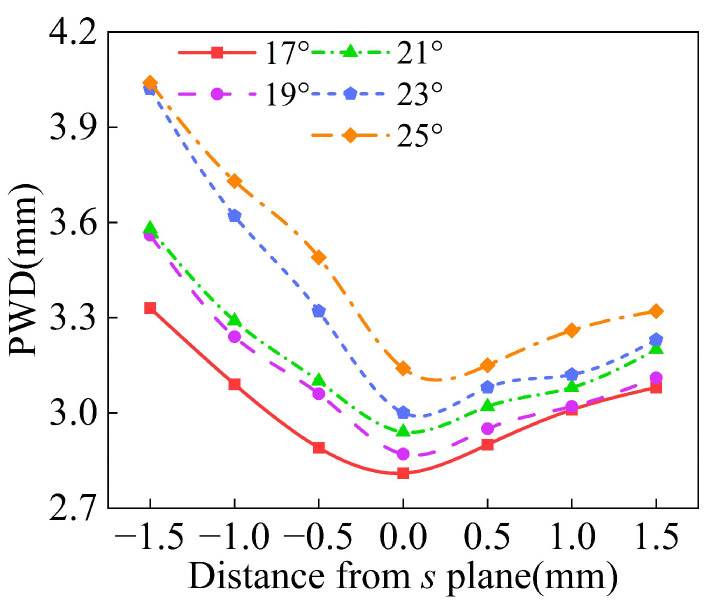
The waist diameter of powder stream at different positions along Z axis with different inclinations *φ*.

The process of powder convergence can be divided into three regions: the pre-waist region, the waist region, and the dispersion region. In L-DED, the distance between the powder convergence plane and the deposition surface is no more than 1.5 mm, so the waist diameter distribution of different powder feeding inclination angles *φ* is analyzed at 1.5 mm above and below the convergence plane *s*. As shown in Figure 10, the *s* plane in the figure is parallel to the bottom plane of the annular nozzle and 10 mm away from it. It is assumed that the vertical downward direction along the Z axis is the positive direction. With the increase in powder feeding inclination angle *φ*, the difference in the waist diameter at the same distance above and below the convergence plane becomes larger, indicating a smaller the waist region. Therefore, when the powder feeding inclination *φ* is 17°, the best powder convergence effect is obtained.

#### 4.1.3. Outlet Gap

The outlet gap *d*_oc_ used by the coaxial annular nozzle is too narrow, which can easily lead to blockage in the powder delivery channel. The simulation model keeps the parallel outlet structure and powder feeding inclination angle *φ* of 17° unchanged, and the size values of outlet gap *d*_oc_ are set to 1 mm, 0.7 mm, and 0.5 mm, respectively, to analyze the influence of outlet gap *d*_oc_ on powder aggregation.

**Figure 11:**
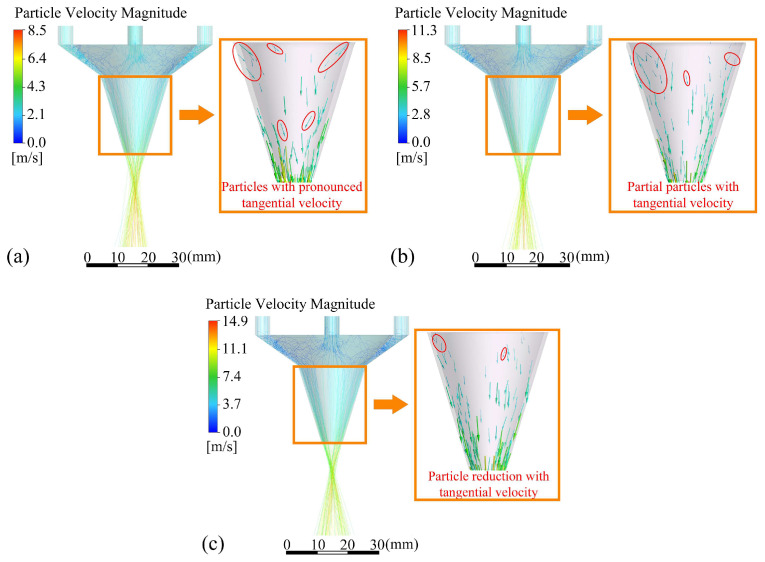
Particle trajectories and velocity vectors of powder with different outlet gaps(the vectors of powder particles with obvious tangential velocity are circled in red circles in the figure): (**a**) 1 mm, (**b**) 0.7 mm, and (**c**) 0.5 mm.

Particle trajectories and velocity vectors of particles entering different outlet gaps *d*_oc_ are shown in Figure 11. The smaller the *d*_oc_, the smaller the tangential velocity vector the powder particle, the lower the number of particle trajectory deviations, and the better the convergence of the powder particles after ejection from the annular nozzle. In addition, the decrease in *d*_oc_ also leads to an increase in the speed of powder particles. The maximum speed of powder particles with *d*_oc_ of 0.5 mm is increased by 75.3% compared with that with *d*_oc_ of 1 mm. The powder particles still maintain high speed after leaving the nozzle, increasing the ability of powder particles to resist the interference of laser shielding gas and helping to ensure the stability of convergence.

**Figure 12:**
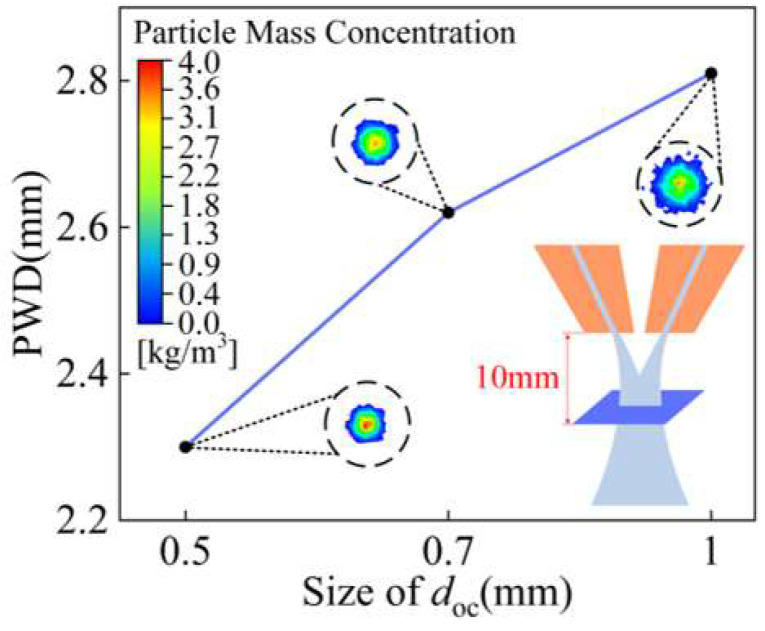
The waist diameter of powder stream with different outlet gaps *d*_oc_.

**Figure 13:**
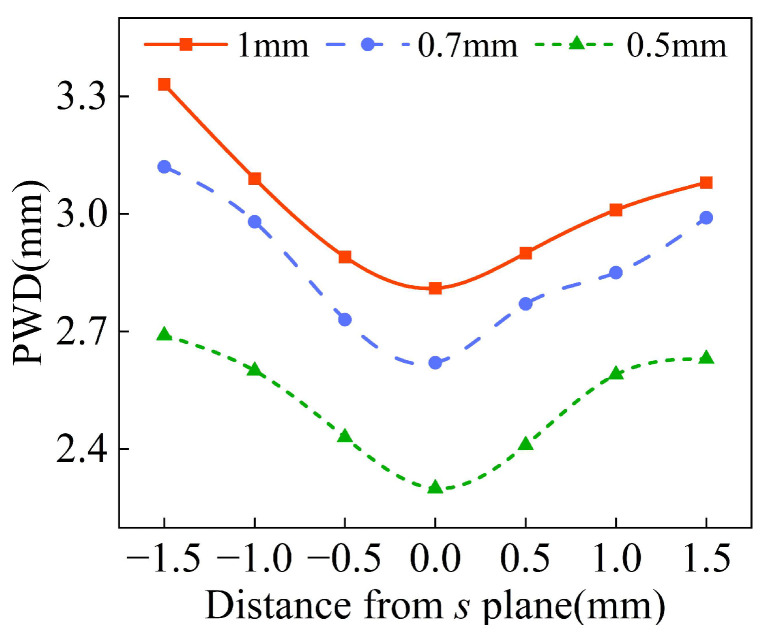
The waist diameter of powder stream at different positions along Z axis with different outlet gaps *d*_oc_.

The powder waist diameter of *d*_oc_ with different outlet gaps at the convergence plane is shown in Figure 12. When *d*_oc_ is 0.5 mm, the powder waist diameter is 2.3 mm, which decreases by 18% compared with that when *d*_oc_ is 1 mm. As shown in Figure 13, with the decrease in *d*_oc_, the difference in waist diameter is not significant at the same distance above and below the convergence plane and the better the powder convergence.

#### 4.1.4. Inlet Shape

Upon the aforementioned analysis, it is evident that at *d*_oc_ of 0.5 mm, there still exists a minor portion of powder particles with significant tangential velocity. Therefore, the structure of the OS of the annular nozzle is not enough to weaken the tangential velocity component of the powder particles, and selecting the appropriate inlet shape structure is also an important way to reduce the tangential velocity of the powder particles and improve the powder convergence. In order to investigate the influence of different inlet shapes on powder aggregation, four different inlet shapes as shown in Figure 3 are set as the IS of the annular nozzle, and the parallel type of outlet structure, powder feeding inclination *φ* of 17°, and outlet gap *d*_oc_ of 0.5 mm are taken as the structural parameters of the OS. By analyzing the influence of different inlet shapes on powder aggregation, the optimal inlet shape structure is determined.

**Figure 14:**
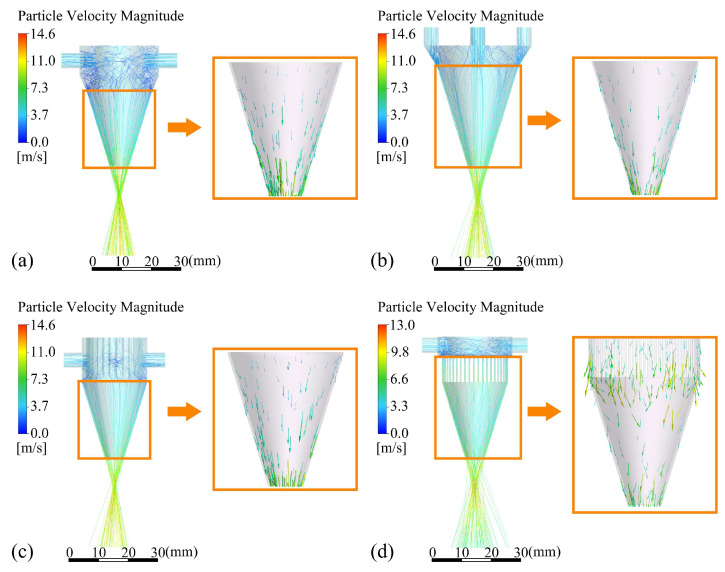
Particle trajectories and velocity vectors of powder with different inlet shapes: (**a**) Case a, (**b**) Case b, (**c**) Case c, and (**d**) Case d.

The powder particles enter four different inlet shapes, which show different convergence states. For the convenience of description, Cases a–d correspond to the four inlet shapes (a)–(d) in Figure 14. The figure shows the trajectory and velocity vector of the powder particles with different inlet shapes. It can be seen from the particle trajectory that the powder convergence of inlet shapes (a) and (c) is better than that of the two other inlet shapes. The structure of the powder storage chamber and the structure of the corrected direct-current groove both play a “drainage” role in the powder particles entering the OS, which helps to reduce the tangential velocity component of the powder particles entering the OS part from the IS part. The structure of the OS part restricts the movement of powder mainly in the feeding tilt direction φ, which greatly unifies the velocity direction of the powder particles after they are emitted from the nozzle and improves the convergence of the powder. However, the inlet shape (b) only extends the structure of the parallel outlet of the OS and lacks a “drainage” structure for the powder particles. Some of the powder particles still enter the OS with a certain tangential velocity, and the OS structure has a weak inhibition effect on the tangential velocity component of the powder particles, resulting in a poor powder convergence effect. The inlet shape (d) is designed with the rectifier tube structure as the “drainage” structure of the IS. The powder particles should correct the direction of movement in the collision inside the rectifier tube and weaken the tangential velocity of the powder particles. However, in order to ensure the uniform distribution of powder particles inside the nozzle, the incident direction should be set off from the axis. Instead, it causes more powder particles to enter the OS with higher tangential velocity, which aggravates the dispersion phenomenon of powder particles after ejection.

**Figure 15:**
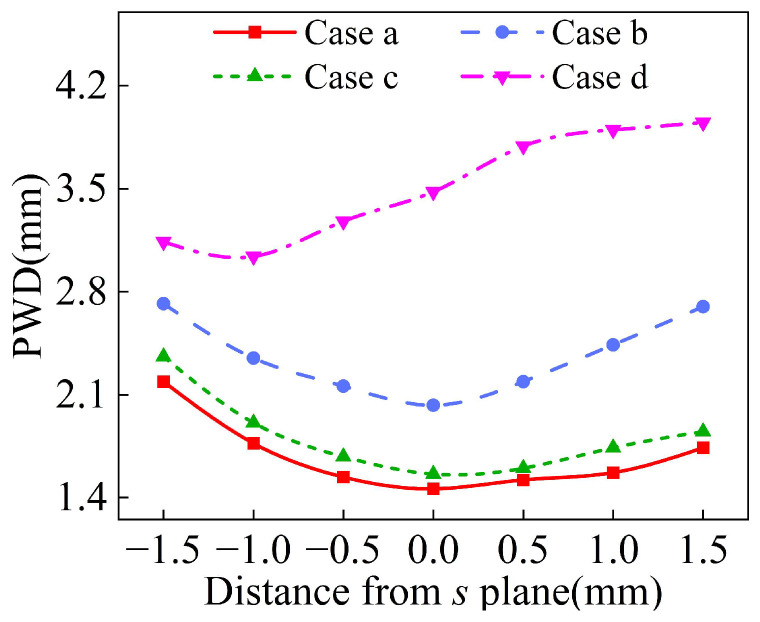
The waist diameter of powder stream with different inlet shapes.

**Figure 16:**
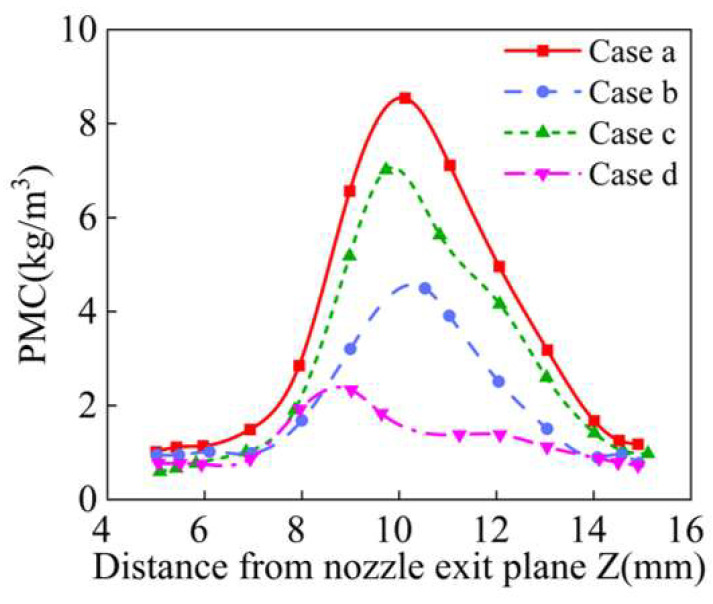
The powder mass concentration at different positions along Z axis with different inlet shapes.

The powder waist diameter and powder mass concentration distributions of the powder bundle with different inlet shapes along the Z axis are shown in Figure 15 and Figure 16. Among the four inlet shapes analyzed, Case a obtained the best powder convergence, and its powder waist diameter at the convergence plane *s* is 1.42 mm, which is 38% lower than that of the inlet shape before optimization. On the contrary, in Case d, the convergence becomes worse. The “drainage” rectifier structure is designed mainly to weaken the tangential velocity of powder particles. If the length of the rectifier tube is too small or the diameter is too large, the collision frequency of powder particles will be reduced, it is difficult to dissipate the tangential velocity of powder particles, and ultimately the powder convergence is worse. Therefore, the inlet shape with a powder storage chamber structure is the best geometrical characteristic structure among the four analyzed, which is conducive to improving the powder utilization rate of the annular nozzle.

### 4.2. Effect of Particle Size on Powder Convergence

In L-DED, the commonly used powder particle size range is 15–150 μm, and the powder particle size range is different for different nozzle types. In order to investigate the influence of different particle size ranges on the powder aggregation of annular nozzles, four sets of simulation models with different particle size ranges are designed and established, which are 15–53 μm, 53–90 μm, 90–120 μm, and 120–150 μm, respectively. By analyzing the distribution of powder mass concentration in the convergence plane of different particle size ranges, the law of influence of particle size range on powder convergence is obtained.

**Figure 17:**
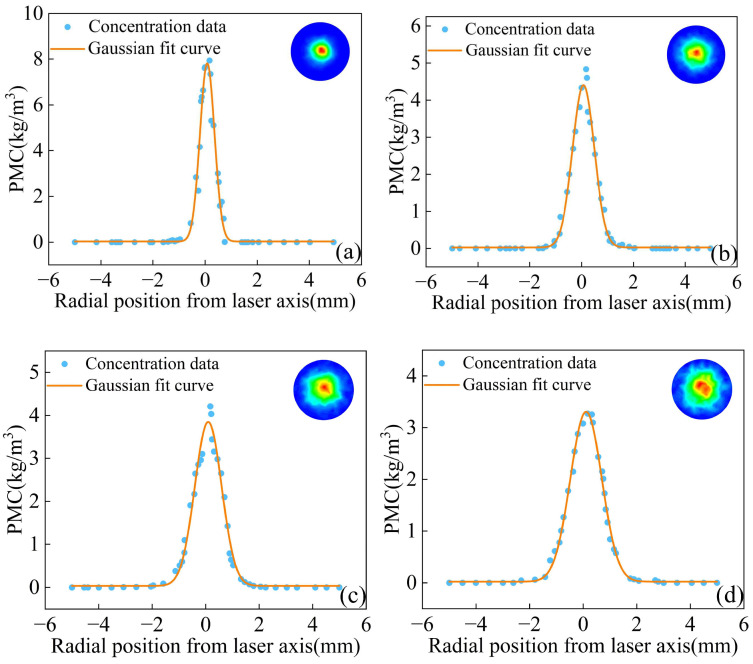
The function fitting of the powder mass concentration and cloud diagram of concentration distribution: (**a**) 15–53 μm, (**b**) 53–90 μm, (**c**) 90–120 μm, and (**d**) 120–150 μm.

**Figure 18:**
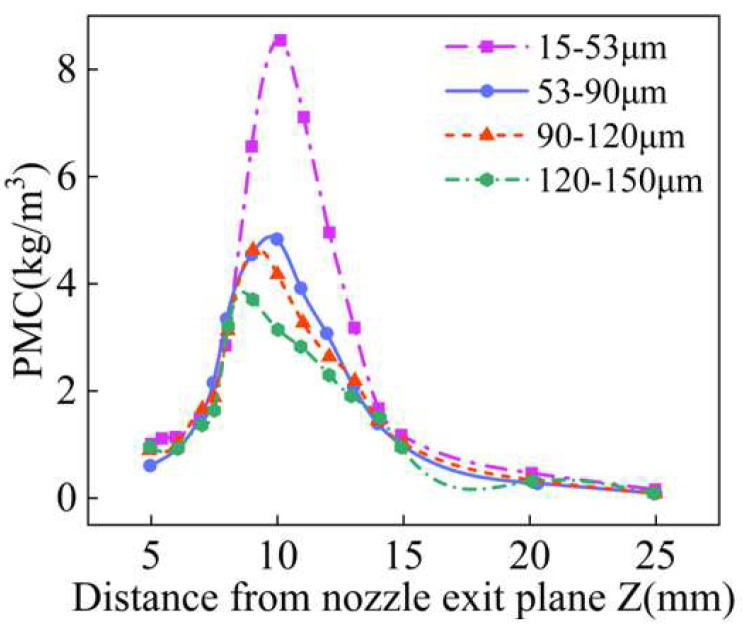
The powder mass concentration at different positions along Z axis with different powder size ranges.

After powder aggregation, its mass concentration can be approximated as a Gaussian distribution in the radial direction. Figure 17 shows the Gauss fitting concentration distribution curve of different powder particle sizes at the convergence plane, and the powder concentration distribution at the corresponding convergence plane is shown in the upper right corner. With the increase in powder particle size, the maximum powder mass concentration of the convergence plane gradually decreased, and the waist diameter gradually increased. When the powder particle size is 120–150 μm, the waist diameter is 2.58 mm, which increased by 81.7% compared with the waist diameter of particles of 15–53 μm. In addition, as the particle size of the powder increases, the interaction force between the powder particles increases, resulting in the deterioration of the fluidity of the powder and the shift of the distribution position of the maximum mass concentration of the powder stream. As shown in Figure 18, with the increase in the particle size of the powder, the position of the maximum mass concentration of the powder flow along the Z axis shifts upward. Therefore, a small particle size is more suitable for annular nozzles, which is conducive to improving the powder utilization rate in the L-DED process and improving the forming efficiency and forming quality of the additive manufacturing parts.

### 4.3. Effect of Gas Rate on Powder Convergence

For the L-DED powder particles driven from the coaxial annular nozzle into the molten pool by the carrier gas, the carrier gas plays an indispensable role in powder transmission, while the coaxial laser shielding gas effectively prevents melted powder particles from rebounding and damaging the optical lens. To investigate the impact of the coaxial laser shielding gas and carrier gas on powder aggregation, simulations were conducted using powder particles ranging in size from 15–53 μm. The results analyzed different flow rates of coaxial laser protective gas and carrier gas to determine their influence on powder aggregation.

**Figure 19:**
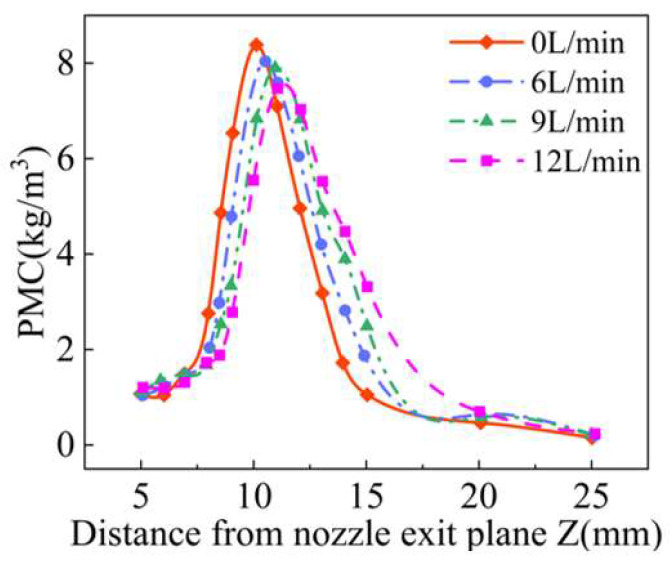
The powder mass concentration at different positions along Z axis with different flow rates of the laser shielding gas.

**Figure 20:**
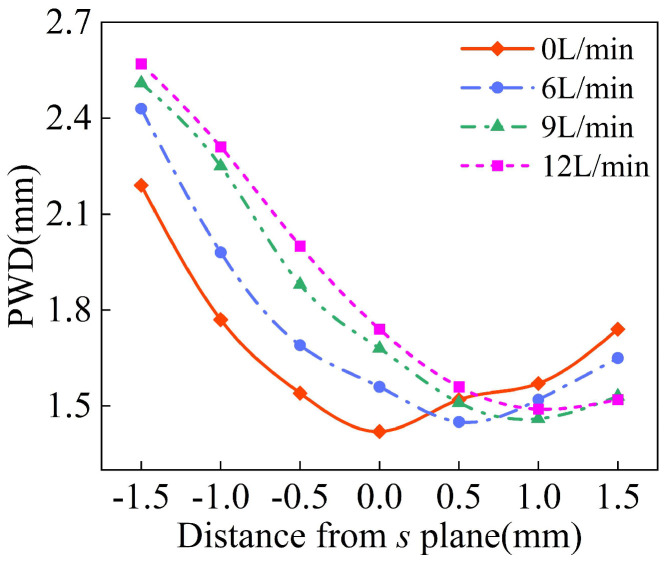
The waist diameter of powder stream at different positions along Z axis with different flow rates of the laser shielding gas.

As shown in Figure 19, the changes of the longitudinal convergence of powder produced by four groups of laser protective gas with different flow rates were analyzed, and the carrier gas flow rate of each group was set to 3 L/min. With the gradual increase in the laser shielding gas flow, the powder mass concentration along the Z axis of the powder stream decreases. Moreover, the addition of the laser shielding gas also increases the speed of the powder particles along the Z axis, so that the position where the highest concentration of the powder flow is also moving down and, when the flow rate of laser shielding gas is 12 L/min, the highest concentration position along the Z axis decreased by about 1 mm. As shown in Figure 20, showing the waist diameter of the powder stream at different positions of the Z axis, corresponding to Figure 19, the waist diameter of the minimum powder stream gradually decreases, but when the flow rate of the laser shielding gas is the highest, the waist diameter of the powder stream is only increased by 3%, indicating that the laser protection gas has little influence on the waist diameter of the powder stream.

**Figure 21:**
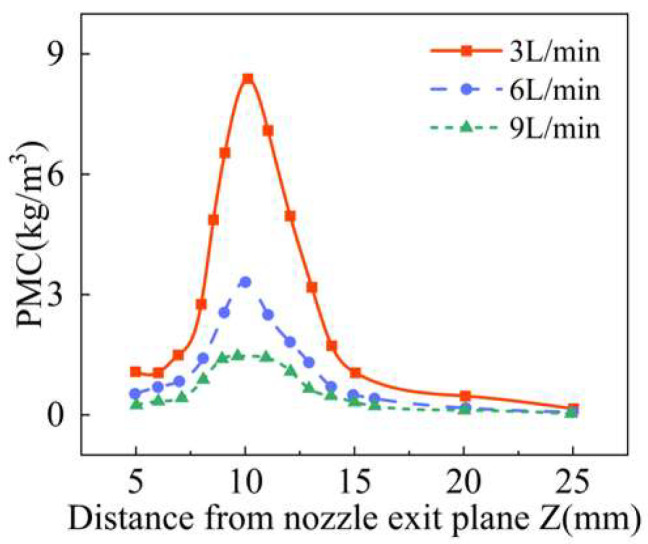
The powder mass concentration at different positions along Z axis with different flow rates of the carrier gas.

**Figure 22:**
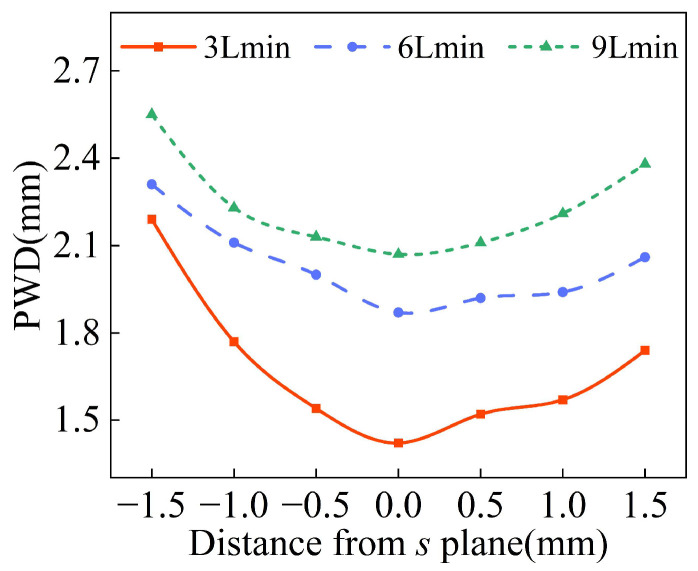
The waist diameter of powder stream at different positions along Z axis with different flow rates of the carrier gas.

To reduce the influence of laser shielding gas input on the analysis results, the flow rate of laser shielding gas is set to 0 L/min. The influence of different carrier gas flow rates on powder aggregation is shown in Figure 21 and Figure 22. As the carrier gas speed increases, the force of the air flow on the powder increases, resulting in higher kinetic energy after the powder leaves the nozzle. However, due to the limited outlet gap *d*_oc_ constraint on the powder particles, the powder particles with higher velocity shift a longer distance before reaching the convergence plane *s*, resulting in an increase in the waist diameter of the powder stream and a rapid decrease in the powder mass concentration after corresponding convergence. Compared with the influence of laser shielding gas on powder aggregation, the impact of carrier gas flow rate is more significant. When the carrier gas flow rate is 9 L/min, the waist diameter of the powder bundle increases by 46%. Therefore, selecting the appropriate carrier gas flow rate is needed to avoid powder dispersion.

Finally, the optimized structural parameters of the annular nozzle are shown in Table 6. The powder convergence generated by this structure is the best. When the inlet carrier gas velocity is 3 L/min, the powder feeding rate is 3 g/min, the powder particle size range is 15–53 μm, and the flow rate of the laser shielding gas is 0 L/min, the diameter of the powder waist after convergence is 1.42 mm.

## 5. Conclusions

In order to improve the forming quality of L-DED additive parts and the powder utilization rate for the annular nozzle, in this paper, the finite element analysis model of an annular nozzle with a powder feeding channel is established, and corresponding verification experiments are designed to verify the effectiveness of the analysis method and model. The influence of the annular nozzle powder delivery channel, powder particle size, and carrier gas velocity on powder convergence is considered, and the influences of different characteristic parameters on powder convergence are obtained. The waist diameter of the converged powder stream is reduced. The results show that, among the three annular nozzle outlet structures, the parallel type of outlet structure obtains the minimum waist diameter and ensures the approximate Gaussian distribution of convergent powder concentration. In addition, with the decrease in powder feeding inclination angle *φ* and outlet gap *d*_oc_, the waist diameter of the powder stream gradually decreases. When the powder feeding inclination *φ* is 17° and the outlet gap *d*_oc_ is 0.5 mm, the optimal structural parameters of the annular nozzle output section are obtained. The inlet shape with the powder storage chamber structure in the input section greatly reduces the tangential velocity component of the powder particles in the way of “drainage” and obtains the best powder convergence effect, and the final powder waist diameter is 1.42 mm. Moreover, with the increase in powder particle size, the waist diameter of the powder stream is significantly increased by 82% for the annular nozzle. The maximum powder mass concentration distribution shifts upward along the Z axis due to the poor powder fluidity. With the increase in carrier gas velocity and laser shielding gas velocity, the powder mass concentration decreases gradually, and the waist diameter of the powder stream increases. But the laser shielding gas also affects the aggregation position of the powder in the Z direction. The research provides a theoretical basis for an L-DED annular nozzle’s structural optimization.

Although this paper systematically analyzes the individual effects of factors such as the powder feed channel structure, powder particle size range, carrier gas velocity, and laser shielding gas velocity in the annular nozzle on powder convergence, there are still many dynamic process parameters that cannot be neglected in actual production, such as powder feeding rate, powder material, nozzle working angle, etc. In addition, the model constructed in this paper ignores the influence of the thermal field on powder, the internal mechanism of laser–powder interaction in the process of laser directional deposition has not been deeply explored, and there is a lack of theoretical analysis of this process. Future research can build multiphysics coupling models to systematically study the thermal fields, the flow fields, and the laser–powder interaction mechanisms.

## Figures and Tables

**Table 1:** Nozzle structure parameter.

Parameters	a	b	s	*φ*	*d*_uc_	*d*_oc_
Values	16 mm	25 mm	10 mm	25°	1 mm	1 mm

**Table 2:** The parameters used in the CFD model.

Parameter	Values
Gas density (kg/m^3^)	1.78
Molecular viscosity (kg·m^−1^·s^−1^)	2.125 × 10^5^
Carrier gas inlet velocity (L/min)	3
Powder material	TC4
Powder density (kg/m^3^)	4500
Powder feeding rate (g/min)	3

**Table 3:** Powder particle parameter.

Min Diameter (μm)	Max Diameter (μm)	Average Diameter (μm)	Spread Parameter	Number of Diameters
15	53	35	3.5	10

**Table 4:** Experimental nozzle structure parameter.

Parameters	a	b	s	*φ*	*d*_uc_	*d*_oc_
Values	17.5 mm	25 mm	10 mm	24°	0.5 mm	0.5 mm

**Table 5:** *d*_uc_ and *d*_oc_ size parameters.

.	Case 1	Case 2	Case 3	Case 4
*d*_uc_/mm	3	2	1	2
*d*_oc_/mm	1	1	1	1

**Table 6:** Optimized annular nozzle powder feeding structure parameters.

	Input Section	Output Section		
Optimal Parameters	Input shape	Outlet shape	Feeding inclination angle *φ*	Outlet gap *d*_oc_
Values	Powder storage chamber structure	parallel type	17°	0.5 mm

## Data Availability

The original contributions presented in this study are included in the article. Further inquiries can be directed to the corresponding author.
